# A high performance self-powered photodetector based on a 1D Te–2D WS_2_ mixed-dimensional heterostructure†

**DOI:** 10.1039/d1na00073j

**Published:** 2021-03-15

**Authors:** Lixiang Han, Mengmeng Yang, Peiting Wen, Wei Gao, Nengjie Huo, Jingbo Li

**Affiliations:** School of Materials and Energy, Guangdong University of Technology Guangzhou 510006 China; Institute of Semiconductors, South China Normal University Guangzhou 510631 P.R. China gaowei317040@m.scnu.edu.cn njhuo@m.scnu.edu.cn

## Abstract

One-dimensional (1D)–two-dimensional (2D) van der Waals (vdWs) mixed-dimensional heterostructures with advantages of an atomically sharp interface, high quality and good compatibility have attracted tremendous attention in recent years. Herein, a mixed-dimensional vertical heterostructure is constructed by transferring mechanically exfoliated 2D WS_2_ nanosheets on epitaxially grown 1D tellurium (Te) microwires. According to the theoretical type-II band alignment, the device exhibits a photovoltaic effect and serves as an excellent self-powered photodetector with a maximum open-circuit voltage (*V*_oc_) up to ∼0.2 V. Upon 635 nm light illumination, the photoresponsivity, external quantum efficiency and detectivity of the self-powered photodetector (SPPD) are calculated to be 471 mA W^−1^, 91% and 1.24 × 10^12^ Jones, respectively. Moreover, the dark current of the SPPD is highly suppressed to the sub-pA level due to the large lateral built-in electric field, which leads to a high *I*_light_/*I*_dark_ ratio of 10^4^ with a rise time of 25 ms and decay time of 14.7 ms. The abovementioned properties can be further enhanced under a negative bias of −2 V. In brief, the 1D Te–2D WS_2_ mixed-dimensional heterostructures have great application potential in high performance photodetectors and photovoltaics.

## Abstract 2

Mechanically exfoliated 2D WS_2_ nanosheets were transferred on epitaxially grown 1D tellurium microwires. The 1D Te–2D WS_2_ heterostructures have potential in self-powered photodetectors and photovoltaics.

## Graphical abstract

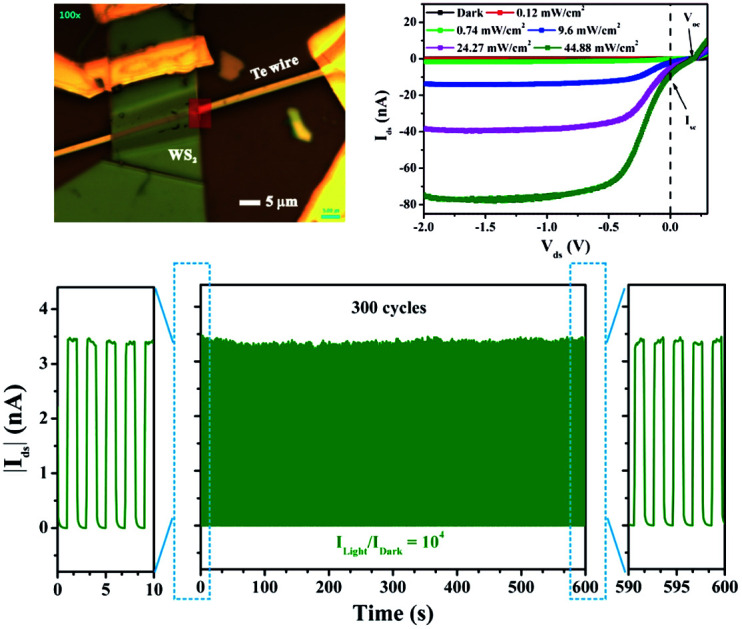

## Introduction

Due to the quantum confinement effect and the strong interlayer coupling effect, two-dimensional (2D) layered materials such as transition metal dichalcogenides (TMDs) (molybdenum disulfide, tungsten disulfide, *etc.*) have attracted tremendous attention with unique thickness dependent and strain-tunable physical properties.^1–7^ In recent years, beyond the discovery of graphene, other novel monoelemental 2D layered materials such as black phosphorus (BP), arsenic (As), bismuth (Bi), tellurium (Te), and antimonene (Sb), which show a tunable band gap, high theoretical carrier mobility, atomically flat surface, strong spin orbital torque, and high light absorption efficiency, have been experimentally explored as promising candidates for applications in field effect transistors (FETs), spintronics and photodetectors (PDs).^8–11^ Among them, tellurium, a quasi-2D semiconductor with a theoretical band gap of ∼0.35 eV in bulk and ∼1 eV in monolayers, has a trigonal crystal structure. Distinctively, it possesses a number of 1D helical chains of Te atoms stacked together *via* weak van der Waals (vdWs) forces along the *c*-axis, leading to the mixed formation type of wires and nanosheets.^12^ Experimentally, Te exhibits excellent properties such as a recorded high hole mobility (∼700 cm^2^ V^−1^ s^−1^), remarkable air stability (over two months), pristine anisotropic structure (anisotropic ratio of mobility ∼1.43) and broadband absorption spectrum (520 nm to 3.39 μm), which make it a potential candidate for future electronics and optoelectronics.^13–17^

Recently, mixed-dimensional heterostructures like zero-dimensional (0D)–2D, 1D–2D and three-dimensional (3D)–2D have drawn interest from researchers due to their unique properties *via* integrating nanomaterials with different dimensionalities.^18–28^ In 2019, Shang *et al.* demonstrated a p-Se nanotube and n-InSe nanosheet mixed-dimensional vdW heterostructure, which shows a high photocurrent on/off ratio of 10^3^ and a responsivity of 110 mA W^−1^ under zero bias with 460 nm irradiation.^29^ Meanwhile, Li *et al.* reported a strongly coupled mixed-dimensional heterostructure *via* epitaxially grown Te nanowires on MoS_2_. The heterostructure based phototransistors displayed obvious anti-ambipolar transport and rectification behavior as well as a high photoresponsivity of 10^3^ A W^−1^ and a fast response time of 15 ms under 1550 nm communication wavelength.^30^ Above all, a number of research groups have focused on the spintronic, electronic and photo-response properties of Te nanosheets and nanowires,^31–38^ while the photodetection properties of Te microwire based mixed-dimensional heterostructures have rarely been reported. Noticeably, the large dark current and ultrafast electron–hole recombination rate of Te are the main disadvantages for further application because of the narrow band gap of Te in bulk. Fortunately, as a typical TMD material, WS_2_ shows merits of moderate bandgap (1.4–2.0 eV), high optical quality and broadband light absorption coefficiency, which make it an ideal candidate in type-II heterostructure based devices.^39^

In this paper, we demonstrate a mixed-dimensional heterostructure of 1D Te microwires covered by 2D WS_2_ nanosheets *via* a polyvinyl alcohol (PVA) dry transfer method. A built-in electric field forms at the heterojunction interfaces, which can efficiently accelerate the separation of the photogenerated electron–hole pairs under light illumination and deeply suppresses the dark current as well. The photodetection properties are investigated systematically with and without bias. The high responsivity, high detectivity, fast response time and high *I*_light_/*I*_dark_ ratio of the 1D p-type Te microwire–2D n-type WS_2_ nanosheet mixed-dimensional heterostructure can promote the development of novel monoelemental materials for optoelectronic applications.

## Experimental section

### Preparation of Te microwires

High-quality Te microwires were synthesized using the physical vapor deposition (PVD) method under ordinary pressure. High purity Te powder (99.999%, purchased from Aladdin) was placed in the center of the furnace inside a quartz tube. The SiO_2_/Si substrate was placed in the downstream area. The quartz tube was sealed and flushed for 5 min using a hydrogen (4%)–argon gas mixture under 600 sccm to provide an oxygen-free environment. The mixed gas was turned off during the heating up process. When the furnace was heated up to 500 °C within 8 min, the mixed gas was turned on under 500 sccm to carry high density vapor of Te atoms to the substrate. The gas was tuned off immediately once the growth was finished in 3 min and cooled down in the ambient environment.

### Fabrication of mixed-dimensional heterostructure devices

The Te microwire–WS_2_ heterostructure was constructed *via* the fabricated polydimethylsiloxane (PDMS) (Gel Pak, 17 mil)/PVA (4 g PVA powder dissolved in 21 mL deionized water) assisted transfer method. First, the mechanically exfoliated WS_2_ nanosheet supported by PDMS/PVA (removing water by heating at 50 °C for 10 min) was peeled off from the SiO_2_/Si substrate by tweezers. Next, the ideal WS_2_ nanosheet was precisely transferred to the Te microwires *via* a three-dimensional location adjustment platform equipped with an optical microscope (Shanghai OnWay Technology Co., Ltd). Then, the PDMS film was lifted from the cured PVA film after heating at 90 °C for 4 min. Finally, the PVA film was removed in deionized water at 50 °C for 15 min and the final device was achieved after annealing at 150 °C for 30 min under nitrogen gas. 5/50 nm Ti/Au were used as electrodes realized *via* ultra-violet lithography and electron beam evaporation deposition. The schematic diagram of the device fabrication process is displayed in Fig. S1 (ESI†).

### Characterization and measurements

Optical images were captured using an optical microscope (Motic Moticcam Pro 205A). The thickness of the WS_2_ nanosheet and the surface potential difference of the Te microwire–WS_2_ heterostructure were measured by Atomic Force Microscopy (AFM) combined with Kelvin Probe Force Microscopy (KPFM) (Dimension FastScan, BRUKER Co., Ltd), respectively. Raman and photoluminescence (PL) spectral measurements (NOST TECHNOLOGY Co., Ltd) were performed at room temperature under a 50 W 532 nm laser. The Scanning Electron Microscope (SEM) (EM-30 PLUS, COXEM Co., Ltd) image was directly observed without metal spraying. The electrical and photoresponse properties of the devices were recorded using a semiconductor parameter analyzer system (Keithley Agilent B2902A system) with a three-probe station under ambient conditions. A broadband bromine tungsten lamp was used to provide the broadband incident light from 400 nm to 1200 nm. The lasers including the wavelengths of 532 nm and 635 nm with a spot diameter of 4 mm were used as incident light to measure the photo-response properties of the devices. The response time was extracted through a chopper.

## Results and discussion

**Fig. 1:**
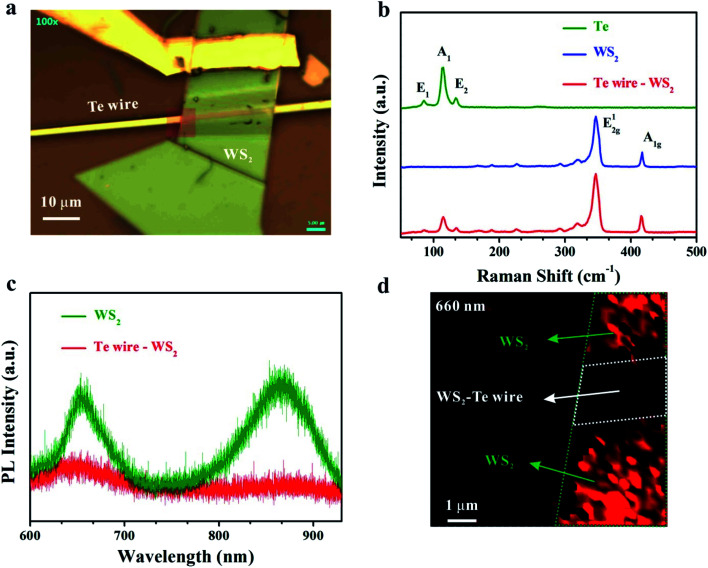
Raman and PL properties of the heterostructure. (a) Optical image of the mixed-dimensional device. (b) Raman spectrum of the Te microwire, WS_2_ and Te microwire–WS_2_. (c) PL spectrum of WS_2_ and Te microwire–WS_2_ under 532 nm laser excitation. (d) PL mapping of the WS_2_ nanosheet on the Te microwire located from the red rectangular region in (a).

As can be seen in Fig. 1a, a chosen thick WS_2_ nanosheet covering on the Te microwire with a width of 2 μm forms a conformal wrapping morphology, giving rise to a mixed-dimensional heterostructure with a large overlapped area.^19^ Fig. S2† shows the SEM image of the device, indicating a smooth surface and the deformation along the region between the flat WS_2_ and Te microwire.

Raman scattering measurement was used to characterize the phonon vibrations and interlayer coupling of the Te microwire–WS_2_ mixed-dimensional heterostructure. Fig. 1b shows the Raman spectrum of the pristine Te microwire, WS_2_ and the heterostructure. The Te microwire shows three vibration peaks located at 85.6 cm^−1^, 115.2 cm^−1^ and 134.3 cm^−1^, which correspond to the in-plane *E*_1_, *E*_2_ and *A*_1_ (out-of-plane) vibrations, respectively.^13^ The in-plane E^1^_2g_ and out-of-plane A_1g_ modes of the multilayered WS_2_ nanosheet are observed at 348.1 cm^−1^ and 418.7 cm^−1^, respectively.^39^ The Raman spectrum of the Te–WS_2_ vdW heterostructure exhibits the combination of phonon modes of both Te wire and WS_2_. Noticeably, the vibration modes of the overlapped Te are weakened. Interestingly, both vibration modes of WS_2_ are enhanced compared to those in the single part, which may be attributed to the strain effect.^40^Fig. 1c shows the PL of the WS_2_ and WS_2_–Te heterostructure with 532 nm laser excitation. In general, the exfoliated multilayered WS_2_ nanosheet shows two clear PL peaks at 660 nm and 861 nm, corresponding to a red-shifted direct optical band gap of 1.88 eV and an indirect band gap of ∼1.44 eV.^39^ A PL quenching effect is observed in the overlapped region for both PL peaks, which is ascribed to a strong interlayer coupling effect between Te and WS_2_. The PL quenching effect indicates that the photo-generated carrier separation process can be significantly accelerated under the designed type-II band alignment. Intuitively, Fig. 1d also displays the corresponding PL mapping image of the WS_2_ on the Te microwire from the rectangular area in Fig. 1a at 660 nm light excitation. The PL intensity of the WS_2_ nanosheet on top of the Te microwire (in the white area) becomes much weaker than that of WS_2_ without Te underneath (in green regions). A similar PL quenching effect is also observed from the PL mapping image under 860 nm light excitation shown in Fig. S3.†

**Fig. 2:**
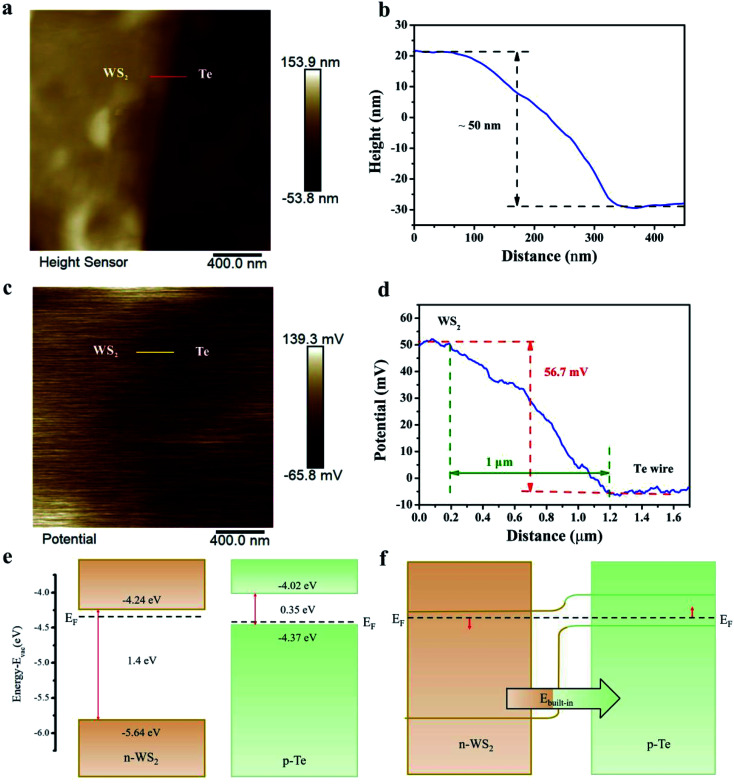
Characterization and band alignment analysis of the mixed-dimensional heterostructure. (a) AFM image of the WS_2_ nanosheet on the Te microwire. (b) The height data of the WS_2_ nanosheet along the scale length of the red line in (a). (c) Spatial potential distribution at the heterostructure interface. (d) The SPD along the yellow line in (c). (e) The energy band diagram of the Te microwire and WS_2_ heterostructure before contact and (f) after contact.

The AFM image of the heterostructure is shown in Fig. 2a. The thickness of the WS_2_ nanosheet is estimated to be 50 nm shown in Fig. 2b. A Kelvin Probe Force Microscope (KPFM) was used to measure the built-in contact potential difference at the interface between the Te microwire and WS_2_. The surface potential distribution (SPD) along the area of the Te microwire, WS_2_ and the AFM tip can be expressed as the following equations:^41^

1 *e*SPD_WS_2__ = *W*_tip_ − *W*_WS_2__

2 *e*SPD_Te_ = *W*_tip_ − *W*_Te_

where *e* is the electronic charge, and *W*_WS_2__, *W*_tip_ and *W*_Te_ are the work functions of the WS_2_, Te microwire and AFM tip, respectively. Then, the Fermi level difference Δ*E*_f_ can be extracted from the above equations:^34^

3 Δ*E*_f_ = *W*_Te_ − *W*_WS_2__ = *e*SPD_WS_2__ − *e*SPD_Te_

Fig. 2c shows the topological image of the SPD of the heterostructure interface. The Δ*E*_f_ and the depletion width along the yellow line are about 56.7 meV and 1 μm from Fig. 2d, which unveils a strong built-in electric field across the WS_2_–Te microwire interface.^42–44^ Moreover, the energy band alignments of the Te microwire and WS_2_ before and after contact are shown in Fig. 2e and f. In general, the indirect bandgaps of multilayered WS_2_ and Te are 1.4 eV and 0.35 eV, respectively. Before contact, the conduction band minima (CBMs) of the WS_2_ and Te microwire are approximately −4.24 eV and −4.02 eV, respectively, and the corresponding valence band maxima (VBMs) of the WS_2_ and Te microwire are approximately −5.64 eV and −4.37 eV, respectively.^39,45^ Δ*E*_f_ is 56.7 meV from the KPFM measurement. Thus, the fabricated Te–WS_2_ heterostructure theoretically has a type-II (staggered gap) band arrangement attributing to the PL quenching effect, which can facilitate the photo-generated carrier generation and separation at the heterointerface.^46^ After contact, the band alignment becomes bent and the electrons and holes can transfer within interlayers *via* a built-in electric field pointing from n-WS_2_ to the p-Te microwire.^47^

**Fig. 3:**
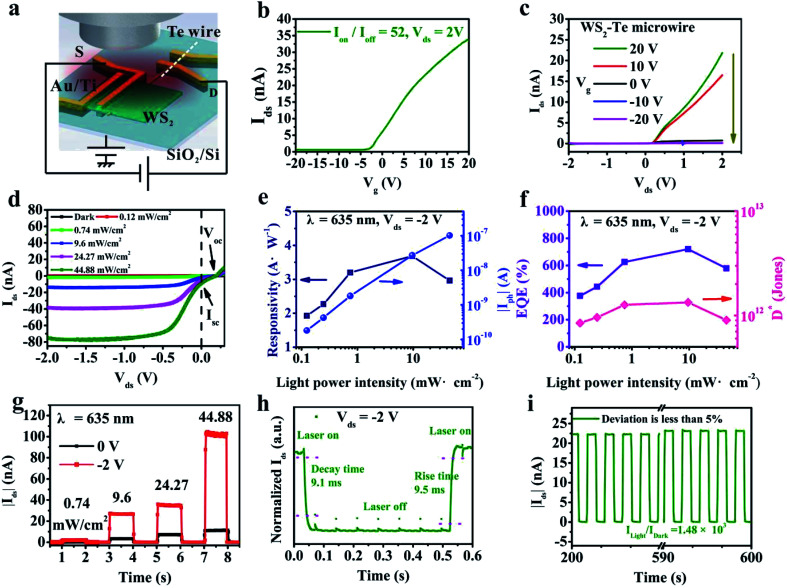
Electronic and optoelectronic properties of the heterostructure based device. (a) The 3D schematic diagram of the mixed-dimensional heterostructure photodetector under 635 nm light illumination. (b) The transfer curve of the device correlated with *V*_g_. (c) Output characteristic curves of the device under *V*_g_ from 20 to −20 V. (d) *I*–*V* curves of the heterostructure device under dark conditions and 635 nm light illumination with various intensities. (e) Photoresponsivity and net photocurrent as a function of light power intensity. (f) EQE and D* dependence on incident light power intensity. (g) Time-dependent photocurrent of the device under different light power intensities at 0 and −2 V bias voltages, respectively. (h) Time-resolved photoresponse measurements. (i) On–off photoresponse with 200 cycles of the heterostructure based photodetector.

The 3D diagram of the 635 nm laser-illuminated Te microwire–WS_2_ heterostructure is shown in Fig. 3a. In Fig. 3b, the mixed-dimensional heterostructure device exhibits n-type (electron dominated) transport behavior, demonstrating that the transport properties of the heterostructure mainly depend on the multilayered WS_2_ channel. Output characteristic curves of the device show that the drain current at a forward bias of 2 V monotonously increases as the *V*_g_ increases, which further confirms the n-type behavior and moderate gate modulation shown in Fig. 3c. The maximum rectification ratio of the device is ∼61 shown in Fig. S4c (ESI†). As a control, the transfer properties of the pristine Te microwire and multilayered WS_2_ nanosheet are shown in Fig. S4a and b (ESI†), where the Te microwire exhibits a strong p-type behavior with a current on/off ratio of ∼1.1 because of the ultra-narrow band gap of 0.35 eV, high conductivity in bulk and strong capacitance screening effect. Meanwhile, the multilayered WS_2_ nanosheet demonstrates a typical moderate n-type behavior with current on/off ratio of ∼10^3^. Fig. 3d demonstrates the *I*–*V* curves of the mixed-dimensional heterostructure device in the range of −2 V to 0.3 V under dark conditions and 635 nm light illumination with various light power intensities. The significantly enhanced current under reverse bias compared to that under forward bias (majority carriers) is shown under light illumination because of the obviously increased minority carriers in the P–N junction. Under higher light power intensity, more photogenerated electron–hole pairs are separated by a built-in electric field and driven by the external reverse bias voltage resulting in the increment of photocurrent. The photovoltaic effect can be seen in Fig. 3d with obvious *V*_oc_ and *I*_sc_, which indicates a well built-in electric field at the interface and will be discussed later.

To further evaluate the photodetection ability of the heterostructure, we calculated the photoresponsivity (*R*_λ_), external quantum efficiency (EQE), detectivity (D*), response time and *I*_light_/*I*_dark_ ratio of the device. In general, *R*_λ_ is used to evaluate the sensitivity of a photodetector, which is defined by the formula^48^4
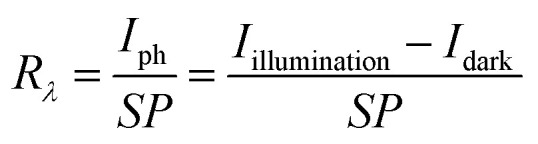
where *λ* is the incident light wavelength, *I*_ph_ is the net photocurrent, *I*_illumination_ is the current under light illumination, *I*_dark_ is the dark current, *P* is the incident light power intensity and *S* is the photo-effective area of the heterostructure (overlapped region of ∼76.2 μm^2^), respectively.

EQE is the ratio of the number of effective photogenerated carriers to the number of incident photons, which can be expressed as^48^5
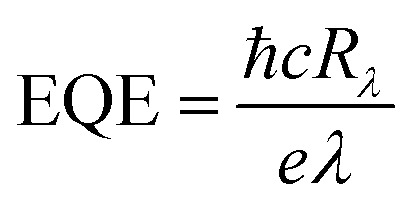
where ℏ is the Planck constant (6.62607015 × 10^−34^ J s), *c* is the velocity of light (3.0 × 10^8^ m s^−1^), *e* is the elementary charge (1.60 × 10^−19^ C) and *λ* is the wavelength of incident light.

Specific detectivity (D*) is an important figure of merit of a photodetector, which shows the ability of a photodetector to detect a weak light signal, as calculated by the following equation:^41^6
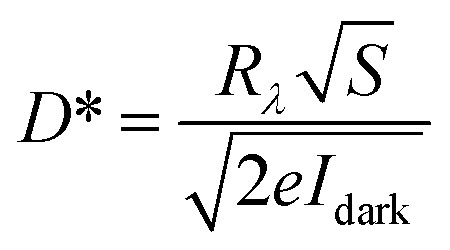


Fig. 3e displays the photoresponsivity and net photocurrent as a function of light power density. The photocurrent increases with increased light power intensity. From the fitting curve which follows a power law of photocurrent and light power intensity (*I*_ph_ ∼ *P*^*α*^), the exponent (*α*) value of 1.07 is obtained shown in Fig. S5 (ESI†). The super-linear behavior may be ascribed to the decrement of Auger recombination sites leading to more photocurrent being transmitted through the pn junction without the trapping effect.^49^ Furthermore, due to the limited trap states, the captured carriers are saturated or reduced under high light power intensity resulting in a significant decrease of the photoresponsivity and increased Auger recombination process.^26^ The maximum value of photoresponsivity reaches 3.6 A W^−1^ at a reverse bias of −2 V shown in Fig. 3e. Fig. 3f demonstrates the dependence of EQE and D* of the mixed-dimensional photodetector on the incident light power intensity. The maximum EQE and D* are 720% and 1.34 × 10^12^ Jones, respectively. Fig. 3g illustrates the time-resolved response behaviors of the mixed-dimensional heterostructure with varying light power intensities at biases of 0 V (in black) and −2 V (in red). With higher light power intensity, more photogenerated electron–hole pairs can contribute to the photocurrent. Under a *V*_ds_ of −2 V, the width of the depletion region is broadened and the corresponding built-in electric field of the heterostructure is enhanced.^44^ Therefore, the separation and collection of photogenerated electron–hole pairs are accelerated resulting in the increment of photocurrent and faster response time. Moreover, response time is one of the important parameters for the photodetector, which is defined as the time obtained from 10–90% (*τ*_rise_ is called rise time) to 90–10% (*τ*_decay_ is called decay time) of the net photocurrent.^48^Fig. 3h illustrates the rise time of 9.5 ms and decay time of 9.1 ms at a bias of −2 V, which are comparable to or faster than that in previously reported 1D–2D mixed-dimensional heterostructures. The high electrical conductance of the Te microwire can contribute to the fast response speed and high responsivity as well in Fig. S4a (ESI†). As shown in Fig. 3i, the *I*_light_/*I*_dark_ ratio of the device is as high as 10^3^ at a bias of −2 V under light power intensity because of the low dark current in the PN junction. Furthermore, the deviation is less than 5% within 200 cycles for switching on–off behavior. In comparison, the *I*_light_/*I*_dark_ ratio of pristine WS_2_ only reaches ∼20 due to a large dark current of around 10^−9^ A under −2 V bias. Meanwhile, the switching on–off curve of the WS_2_ nanosheet based photodetector exhibits poor stability shown in Fig. S4d (ESI†), which is mainly ascribed to the persistent photoconductive (PPC) effect in multilayered WS_2_.^50^ The photoresponse properties of the pristine WS_2_ nanosheet are shown in Fig. S6 and S7 (ESI†), which is worse than that in the heterostructure.

**Fig. 4:**
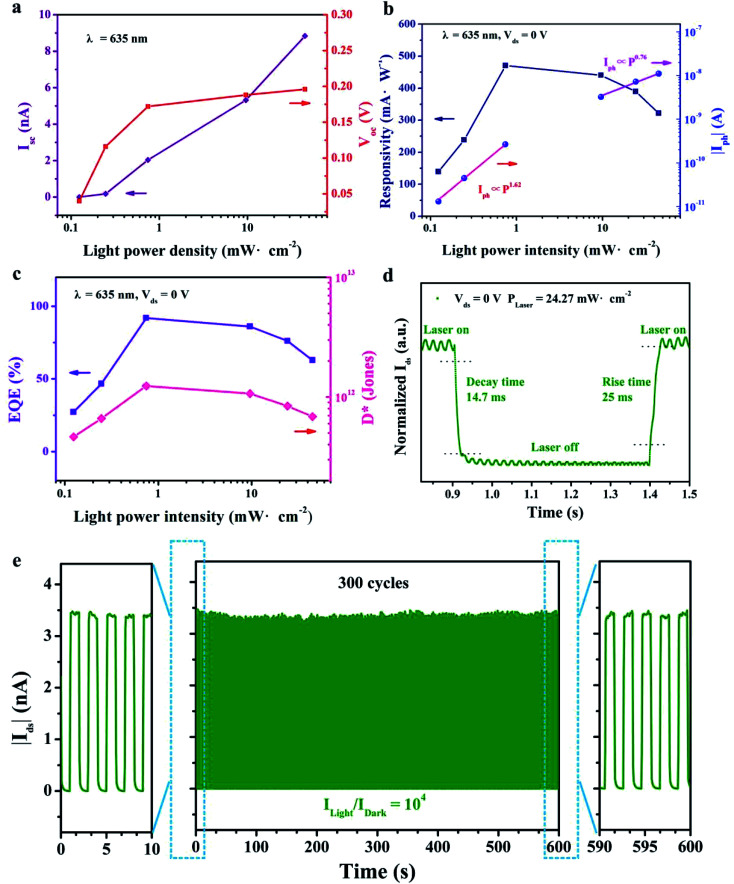
Self-powered photo-response characteristics of the heterostructure device under 635 nm illumination. (a) The *V*_oc_ and *I*_sc_ of the device *versus* illumination power intensity. (b) Photocurrent and *R*_*λ*_ varying with incident intensities under 0 V bias. (c) EQE and D* as a function of the light power intensity. (d) Rise and decay time of the device under 24.27 mW cm^−2^. (e) Photo-response of the photodetector with 300 cycles.

As we know, self-powered photodetectors are extensively desired in the field of wearable electronics and Internet of Things featuring lower power consumption or a self-sustaining wireless sensing network.^51^ Here, the self-powered photo-response properties of the mixed-dimensional heterostructure photodetector are intensively investigated to highlight the contact quality of the PN junction. The open-circuit voltage (*V*_oc_) of the device is induced by the built-in electric field because of the photogenerated holes accumulating at the n-WS_2_ side and electrons accumulating at the p-Te microwire side. Fig. 4a displays the *V*_oc_ and *I*_sc_ as a function of incident light power intensity. With higher light power intensity, the built-in electric field is strengthened leading to the nonlinear increase of *V*_oc_ and the linear enhancement of *I*_sc_ for the device. Fig. 4b shows the dependence of photocurrent and photoresponsivity on incident light power density. The maximum photoresponsivity of the self-powered heterostructure is as high as 471 mA W^−1^ under 0.74 mW cm^−2^. The exponent (*α*) values of 1.62 (under weak light) and 0.76 (under strong light) are obtained by fitting the measured data because of the complex transfer and recombination process of photo-carriers. Fig. 4c shows the incident light power intensity related EQE and D* of the device; the maximum EQE (91%) and D* (1.24 × 10^12^ Jones) are obtained under 0.74 mW cm^−2^, respectively.

The self-driven heterostructure device also exhibits a fast photoresponse time with a rise time of 25 ms and a decay time of 14.7 ms shown in Fig. 4d. Due to the faster speed of recombination of carriers than the generation and transport processes, the decay time is shorter than the rise time.^52^ As shown in Fig. 4e, the *I*_light_/*I*_dark_ ratio is up to 10^4^ at the dark current level of 3.1 × 10^−13^ A. Last but not least, the switching performance of the as-fabricated self-powered photodetector also shows negligible degradation after 300 cycles without obvious deviation. The photodetection parameters of the 1D Te–2D WS_2_ device compared with the previously reported 1D–2D mixed-dimensional heterostructure are shown in Table 1.

**Table 1:** Comparison of parameters based on 1D–2D mixed-dimensional photodetectors

Sample	Wavelength [nm]	*V*_ds_/*V*_g_ [V]	*I*_light_/*I*_dark_	Rise/decay time [ms]	*R*_λ_ [mA W^−1^]	EQE [%]	D* [Jones]	Ref.
ZnO–WSe_2_	520	−5/—	—	50	670	160	—	25
CuO–MoS_2_	570	−2/0	10^3^	34.6/51.9	157.6	157.6 × 10^3^	—	26 and 27
Se–InSe	460	0/0	10^3^	30/37	110	51	—	29
Te–MoS_2_	1550	2/80	10^3^	15/32	10^6^	—	10^12^	30
ZnO–MoS_2_	532	5/0	—	140/8320	350	80.9	—	28
Te–WS_2_	635	−2/0	1480	9.5/9.1	3690	720	1.34 × 10^12^	This work
Te–WS_2_	635	0/0	10^4^	25/14.7	471	91	1.24 × 10^12^	This work

**Fig. 5:**
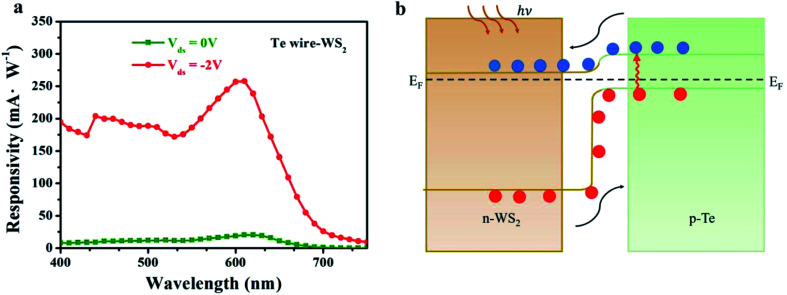
Wavelength-dependent photoresponse performance and the transport mechanism. (a) A broadband spectrum photoresponse behavior of the device. (b) Carrier transport dynamic mechanism of the mixed-dimensional photodetector under light illumination.

In addition, the photoresponsivity spectra of the mixed-dimensional heterostructure device at biases of −2 V and 0 V were also recorded and are shown in Fig. 5a. Notably, the photodetector displays a broadband photo-response ranging from 400 nm to 750 nm wavelength. Obviously, the strongest responsivity peaks are located at an approximately sharp edge of 620 nm under both conditions. The corresponding light power–wavelength diagram is shown in Fig. S8 (ESI†). The broadband photoresponse of the heterostructure device can be attributed to the highly efficient broadband optical absorption spectrum of the WS_2_ nanosheet.^39^ The photodetection properties of the mixed-dimensional heterostructure under 532 nm laser illumination were also investigated shown in Fig. S9 (ESI†). The different photoresponsivity between 532 nm and 635 nm incident light is related to the wavelength-dependence of light absorption and semiconductor energy gap.^48^ The photo-generated carrier transport dynamics mechanism under light illumination is illustrated in Fig. 5b. Under illumination, the photogenerated electron–hole pairs are induced in the depletion between WS_2_ and Te microwire interface. Meanwhile, photocurrent is generated through the separation of photo-generated electron–hole pairs in opposite directions to the metal electrode driven by the built-in electric field with and without external reverse bias voltage.^47^ Meanwhile, the non-radiative recombination rate is reduced within the band structure of the Te microwire.

## Conclusions

In summary, a mixed-dimensional heterostructure based on the 1D Te microwire and 2D WS_2_ nanosheet has been fabricated for high performance photodetectors. Due to the built-in electric field and type-II band alignment, the heterostructure not only shows a fast photoresponse time (*τ*_rise_ = 25 ms, *τ*_decay_ = 14.7 ms) but also an ultralow dark current level of 3.1 × 10^−13^ A compared with the pristine WS_2_ and Te microwire. The photodetection performances of the device under the biases of 0 V and −2 V are higher than or comparable to other reported 1D–2D mixed-dimensional heterostructure based photodetectors. The mixed-dimensional heterostructure can serve as a promising candidate for high performance self-powered photodetectors, promoting the development of 1D–2D hybrid systems for optoelectronic applications.

## Author contributions

W. Gao and J. B. Li designed the project and the experiments; L. X. Han performed the experiments and wrote the paper with help from W. Gao and N. J. Huo; the other authors discussed the results and helped to draw figures.

## Conflicts of interest

There are no conflicts to declare.

## Supplementary Material

NA-003-D1NA00073J-s001
