# Supplementary material for: A high performance self-powered photodetector based on a 1D Te–2D WS2 mixed-dimensional heterostructure
Source: Nanoscale Adv. 2021 Mar 15;3(9):2657–65. doi: 10.1039/d1na00073j (PMC9419060; doi:10.1039/d1na00073j)
Supplement: NA-003-D1NA00073J-s001 [file NA-003-D1NA00073J-s001.pdf]

Electronic Supplementary Material (ESI) for Nanoscale Advances.
This journal is © The Royal Society of Chemistry 2021

High performance photodetector based on 1D Te-2D WS_2 mixed-dimensional heterostructure

Lixiang Han,[a] Mengmeng Yang,[a] Peiting Wen,[b] Wei Gao,*[b] Nengjie Huo*[b] and Jingbo Li[b]

a. School of Materials and Energy, Guangdong University of Technology, Guangzhou, 510006, China

b. Institute of Semiconductors, South China Normal University, Guangzhou 510631, P.R. China

* Corresponding author (email: gaowei317040@m.scnu.edu.cn; njhuo@m.scnu.edu.cn)

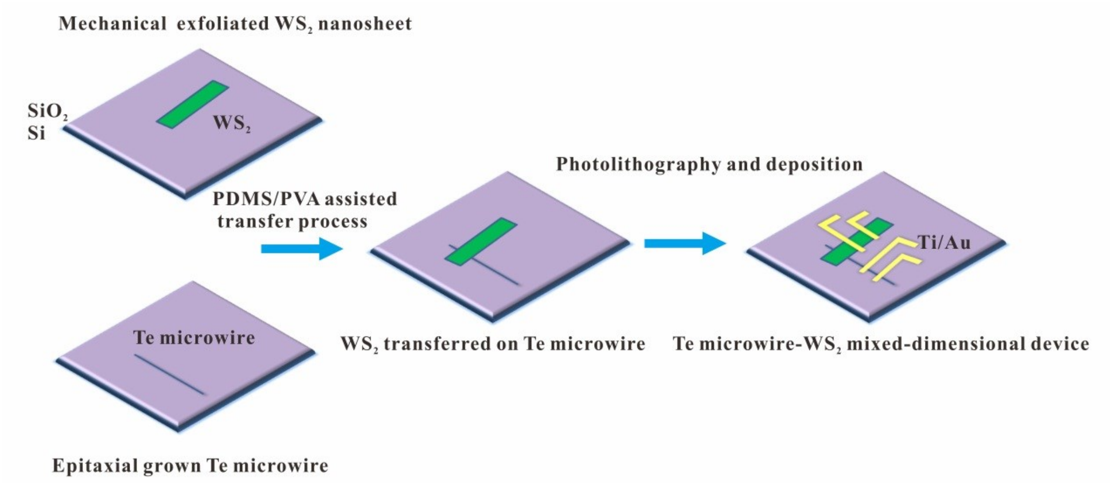

Figure S1. The schematic diagram of the fabrication process of the mixed-dimensional heterostructure device.

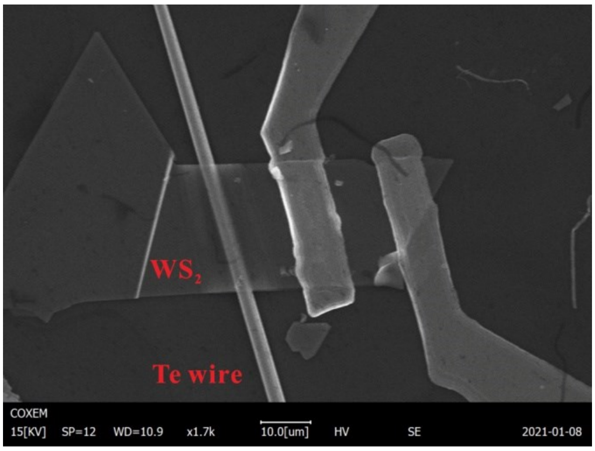

Figure S2. The SEM image of the device.

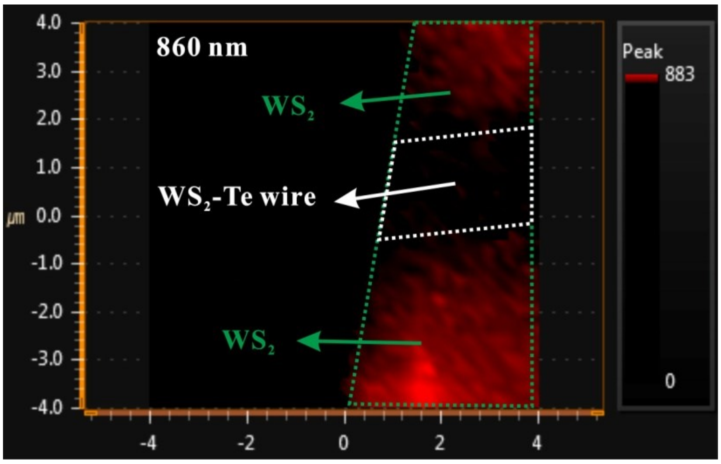

Figure S3. The PL mapping image of the heterostructure under 860 nm light excitation.

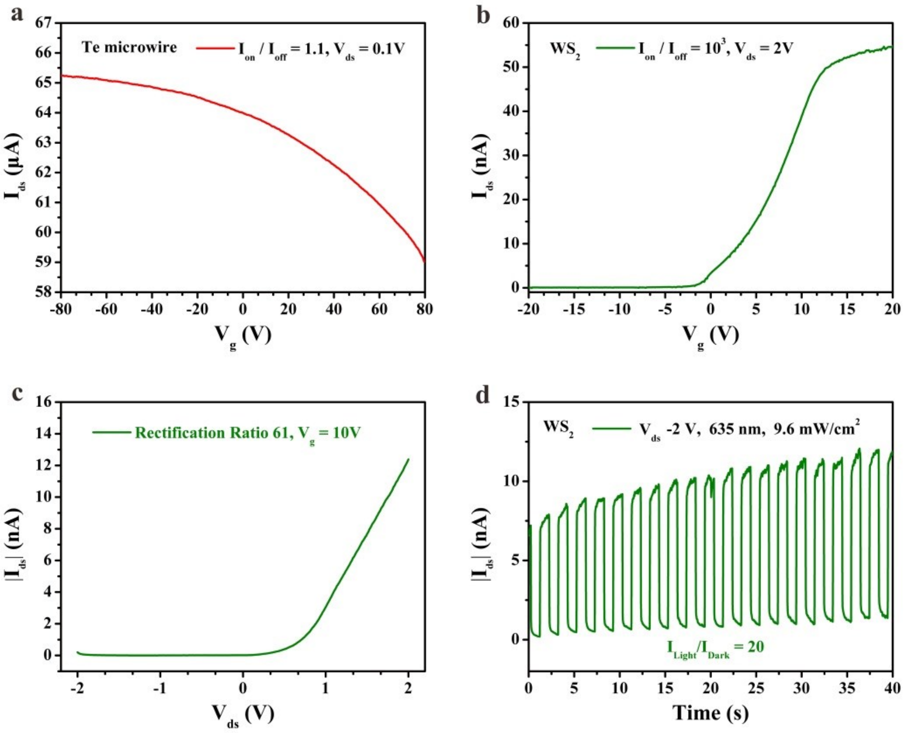

Figure S4. Transfer properties and I-t characteristics. (a) Transfer curve of Te microwire, (b) Transfer curve of WS_2 nanosheet, (c) Rectification curve of the mixed-dimensional heterostructure device, （d）I-t characteristics of WS_2 nanosheet with external bias voltage of -2 V under 635 nm light illumination.

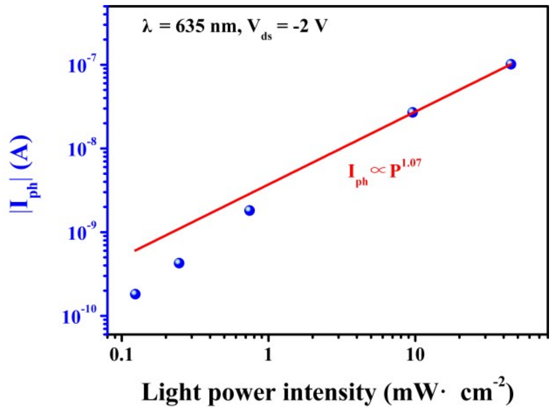

Figure S5. The photocurrent dependent on the light power intensity by a power law of the heterostructure with bias of -2V under 635 nm light illumination.

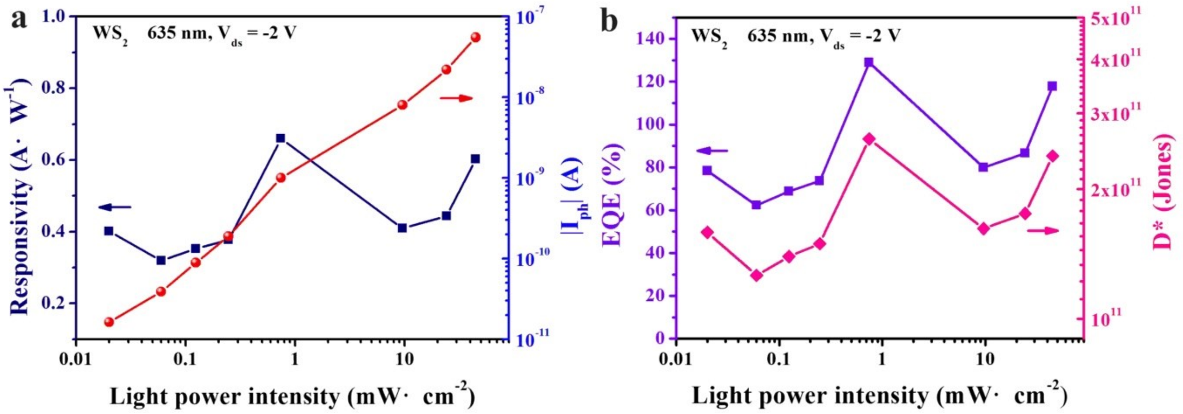

Figure S6. Photoresponse properties of the pristine WS_2 nanosheet under incident laser of 635 nm. (a), (b) demonstrate the photoresponsivity, net photocurrent, EQE and D^* of the WS_2 as a function of light power intensity with V_{ds} of -2V, respectively.

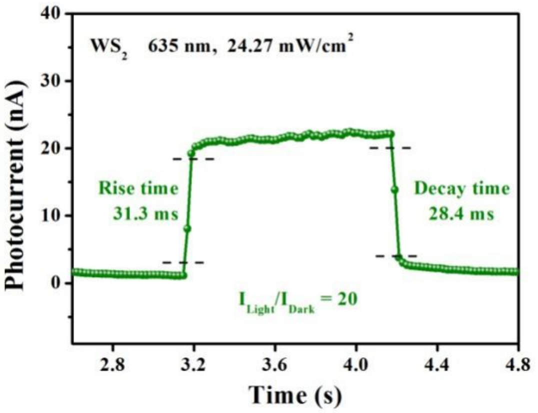

Figure S7. Photoresponse time of the WS_2 nanosheet.

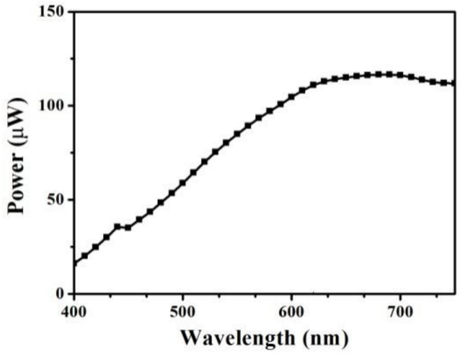

Figure S8. Light power-wavelength diagram.

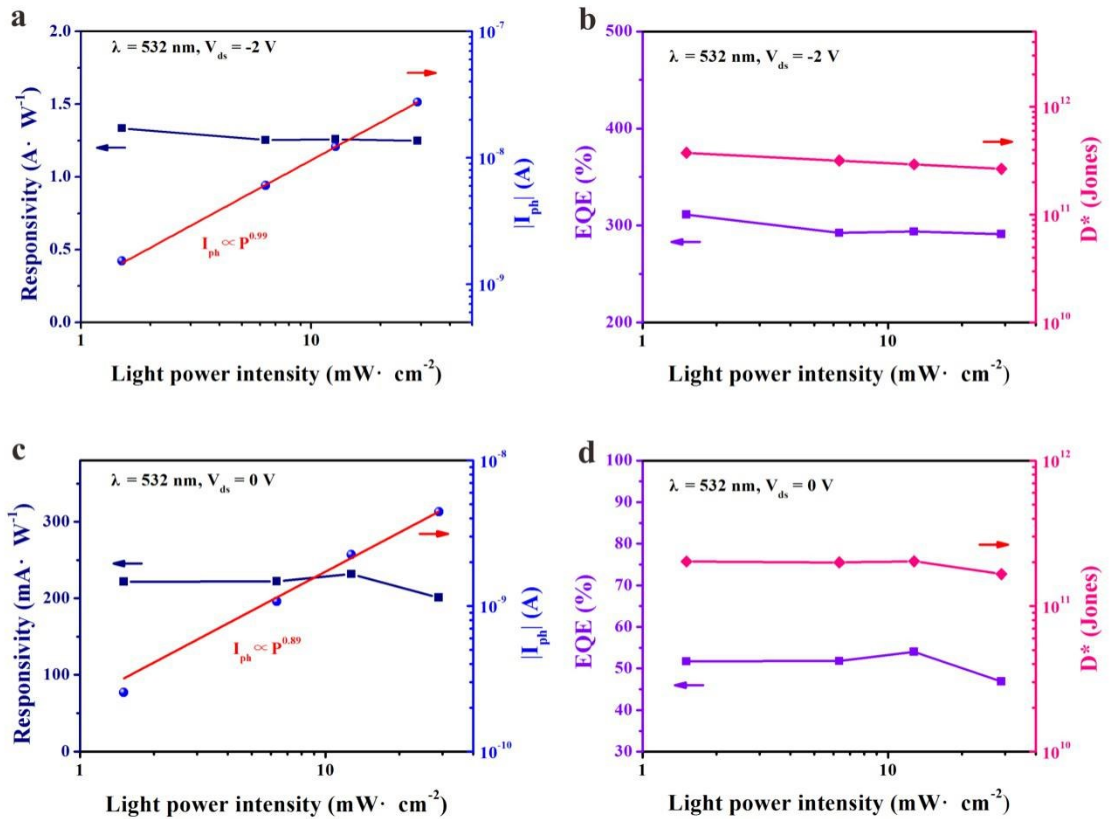

Figure S9. Photoresponse properties of the mixed-dimensional heterostructrue Te microwire and WS_2 nanosheet based photodetector under incident laser of 532 nm. (a), (b) demonstrate the photoresponsivity, net photocurrent, EQE and D^* of the device as a function of light power intensity with external bias voltage V_{ds} of -2V, respectively. (c), (d) display the responsivity, pure photocurrent, EQE and D^* of the device as a function of light power intensity without bias voltage, respectively.
